# Selective Separation of Vanillic Acid from Other Lignin-Derived Monomers Using Centrifugal Partition Chromatography: The Effect of pH

**DOI:** 10.1021/acssuschemeng.1c08082

**Published:** 2022-04-05

**Authors:** Inês L. D. Rocha, André M. da Costa Lopes, Sónia P. M. Ventura, João A. P. Coutinho

**Affiliations:** † CICECO − Aveiro Institute of Materials, Department of Chemistry, University of Aveiro, 3810-193 Aveiro, Portugal; ‡ CECOLAB - Collaborative Laboratory Towards Circular Economy, R. Nossa Senhora da Conceição, 3405-155 Oliveira do Hospital, Portugal

**Keywords:** lignin, aromatic monomers, aqueous biphasic systems, separation, purification, centrifugal partition chromatography

## Abstract

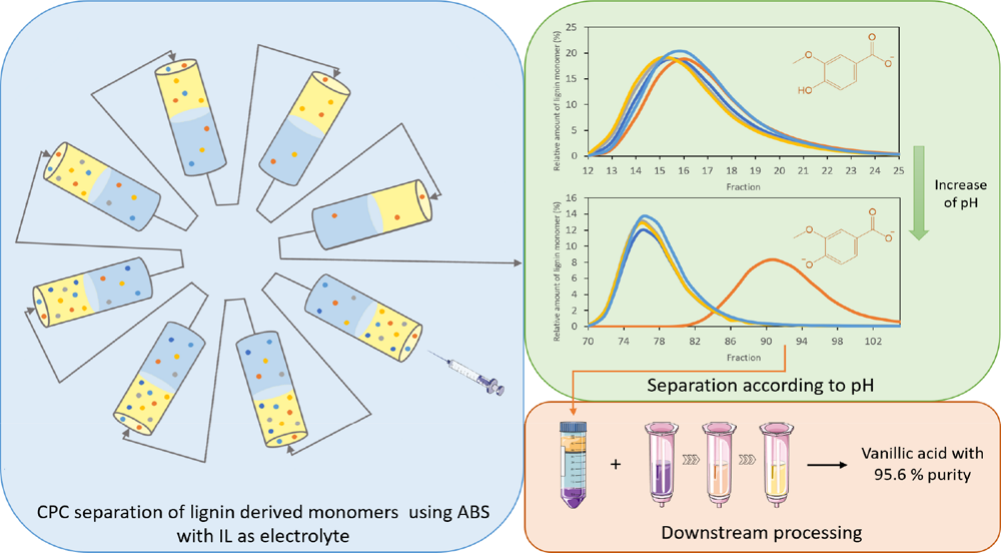

In this work, centrifugal partition chromatography (CPC) assisted by a polyethylene glycol (PEG)/sodium polyacrylate (NaPA) aqueous biphasic system (ABS) was applied in the separation of five lignin-derived monomers (vanillin, vanillic acid, syringaldehyde, acetovanillone, and *p*-hydroxybenzaldehyde). The influence of the system pH (unbuffered, pH 5, and pH 12) and added electrolytes (inorganic salts or ionic liquids (ILs)) on the compound partition was initially evaluated. The obtained data revealed that ILs induced more adequate partition coefficients (*K* < 5) than inorganic salts (*K* > 5) to enable separation performance in CPC, while alkaline conditions (pH 12) demonstrated a positive impact on the partition of vanillic acid. CPC runs, with buffered ABS at pH 12, enabled a selective separation of vanillic acid from other lignin monomers. Under these conditions, a distinct interaction between the top (PEG-rich) and bottom (NaPA-rich) phases of the ABS with the double deprotonated form of vanillic acid is expected when compared to the remaining lignin monomers (single deprotonated). This is an impactful result that shows the pH to be a crucial factor in the separation of lignin monomer compounds by CPC, while only unbuffered systems have been previously studied in the literature. Finally, the recovery of vanillic acid up to 96% purity and further recycling of ABS phase-forming components were approached as a proof of concept through the combination of ultrafiltration and solid-phase extraction steps.

## Abstract 2

Sustainable separation and purification of vanillic acid from other lignin-derived monomers using aqueous biphasic systems coupled with centrifugal partition chromatography were performed.

## Introduction

Among the three major lignocellulosic biomass components, namely, cellulose, hemicelluloses, and lignin, the last has been typically classified as a low-value byproduct, whose major end relies on its combustion.^1^ Although this is a valuable contribution to reduce fossil fuel consumption for energy production, the unique physicochemical properties of lignin offer further perspective toward higher value applications. For instance, the breakdown of the lignin structure is expected to play a major role in the replacement of the aromatic fraction of crude oil for the production of commodities, such as pivotal intermediates in the synthesis of second-generation fine chemicals.^2−4^ Therefore, efficient lignin depolymerization technologies might promote a greener lignin-to-chemical pathway,^5^ enabling the production of heterogeneous mixtures of aromatic compounds. However, technical shortcomings in downstream processing, particularly at the fractionation, isolation, and purification steps of lignin monomers, create a strong technological barrier.

Liquid–liquid separations, combined ionic liquid (IL) and supercritical carbon dioxide extraction (scCO_2_), crystallization, adsorption in specific polymeric resins, and membrane separation are some of the key downstream technologies that have been proposed for lignin monomers (e.g., vanillin, syringaldehyde, and *p*-hydroxybenzaldehyde).^6−11^ However, their low efficiency and selectivity, high cost, and difficult scale-up limit their widespread application in industry. Therefore, the development of more efficient, selective, yet scalable processes toward the separation and purification of lignin-derived monomers is required. An alternative example that has been attracting attention in the past years is centrifugal partition chromatography (CPC).

CPC is a versatile chromatographic downstream technology with the particularity of operating with both (immiscible) stationary and mobile phases in the liquid state. This is a meaningful difference over conventional chromatographic methods, which require a solid stationary phase. Due to the different affinity of the target compounds between each immiscible liquid phase, different elution times can be obtained, resulting in desired compound separation. In this sense, the partition coefficients (*K*) of desired compounds between stationary and mobile phases are important and indicative of expected elution order of compounds in CPC. There is a window of opportunity in CPC separations related to the *K* value of a given compound in a particular solvent system. A biphasic system is considered an ideal medium for separating a desired compound in CPC when the *K* value of such compound is ∼1. In this case, the decision of which phase (upper or lower) will be the mobile or stationary phase is less relevant as the retention volume of the target compound will be very similar in either mode. On the other hand, lower *K* values result in a loss of peak resolution, while high *K* values tend to produce excessive peak broadening and prolonged runs.^12^ According to CPC best practices, only systems with *K* < 5 of target compounds and ideally within the range 0.4 ≤ *K* ≤ 2.5 should be applied for this separation technique. Furthermore, another factor to consider when choosing a biphasic system for CPC separation is the difference between the *K* values of target compounds, which should be maximized to improve the resolution of the separation and prevent co-elution of compounds.^13^ Therefore, CPC provides several advantages over conventional liquid chromatography including high selectivity, lack of irreversible absorption of molecules, high loading capacity, total recovery of injected samples, and easy scale-up. Furthermore, a low solvent consumption, in comparison to conventional processes, makes CPC environmentally friendly, while the liquid nature of the stationary phase prevents problems with cleaning as the no-adsorption phenomenon is observed.

The application of CPC in the fractionation and isolation of phenolic compounds has been studied in the past years using mixtures of organic solvents, such as butanol and water;^14^ ethyl acetate, butanol, and water;^15^ ethyl acetate, ethanol, and water;^16,17^ or quaternary biphasic systems, including an alkane, ethyl acetate, methanol, and water.^18−22^ The latter, also called Arizona biphasic systems, has been coupled with CPC to fractionate and isolate individual compounds from complex mixtures, such as lignans^23^ and lignin monomeric compounds.^22^ For instance, Alherech et al*.* showed the capacity of Arizona L (pentane/ethyl acetate/methanol/water 2:3:2:3 v/v) coupled with CPC to fractionate a complex mixture of lignin-derived monomers resulting from the alkaline aerobic oxidation of lignin.^22^ This approach was able to separate and isolate vanillin and syringic acid, but other compounds like vanillic acid, *p*-hydroxybenzaldehyde, and syringaldehyde were not separated using the Arizona L mixture.^22^ Yet, some environmental concerns can be raised when using Arizona systems since they are solely composed of volatile organic solvents. That is why other biphasic alternatives are being examined for this purpose of lignin monomer separation by CPC.

Over the past decades, aqueous biphasic systems (ABS) have emerged as an alternative to conventional liquid–liquid extraction methods, which traditionally use volatile organic solvents (e.g., Arizona systems).^24^ ABS consist of two immiscible aqueous-rich phases typically based on polymer–polymer, polymer–salt, or salt–salt combinations.^25^ Although both phase-forming components are water-soluble, there is partition into two coexisting phases above a given concentration: a phase rich in one of these compounds, while its counterpart prevails in the opposite phase. Among them, sodium polyacrylate (NaPA) and polyethylene glycol (PEG) have been used as an efficient combination of phase formers originating from polymer–polymer-type ABS capable of purifying multiple biomolecules.^26−28^ In addition, various authors have evaluated the effect of different electrolytes, mainly inorganic salts^28,29^ and, more recently, ILs^26^ and surfactants,^30^ on the improvement of phase separation and biomolecule partition of the NaPA-PEG systems. In this sense, the combination of these two biocompatible, inexpensive, and easy to recycle polymers^28^ is particularly advantageous to couple with CPC. This strategy is expected to upgrade the high level of separation obtained in ABS batch operations into desired continuous processes.^12,31,32^

Up to today, only unbuffered ABS have been studied in CPC separation, including our previous work.^33^ Therefore, the hypothesis that affinity of compounds to both mobile and stationary phases might be affected by ABS pH needs to be evaluated since it may influence the efficiency and the resolution of separation. In this work, the influence of ABS pH (unbuffered, pH 5, and pH 12) and different electrolytes (ILs or inorganic salts) on the partition of several lignin monomers in polymer–polymer-type ABS composed of PEG 8000 and NaPA 8000 was evaluated. The partition of vanillin, vanillic acid, syringaldehyde, acetovanillone, and *p*-hydroxybenzaldehyde, as a representative mixture of a lignin depolymerization stream, was first determined before CPC experiments. The most suitable ABS was selected and coupled with CPC aiming at their efficient separation. Among them, vanillic acid presented distinct physicochemical characteristics with pH variations that enable a major focus on its selective separation and isolation. Further downstream processing combining ultrafiltration (UF) and solid-phase separation (SPE) was also applied to isolate vanillic acid and to recover phase-forming constituents as a proof of concept.

## Experimental Section

### Materials

**Figure 1:**
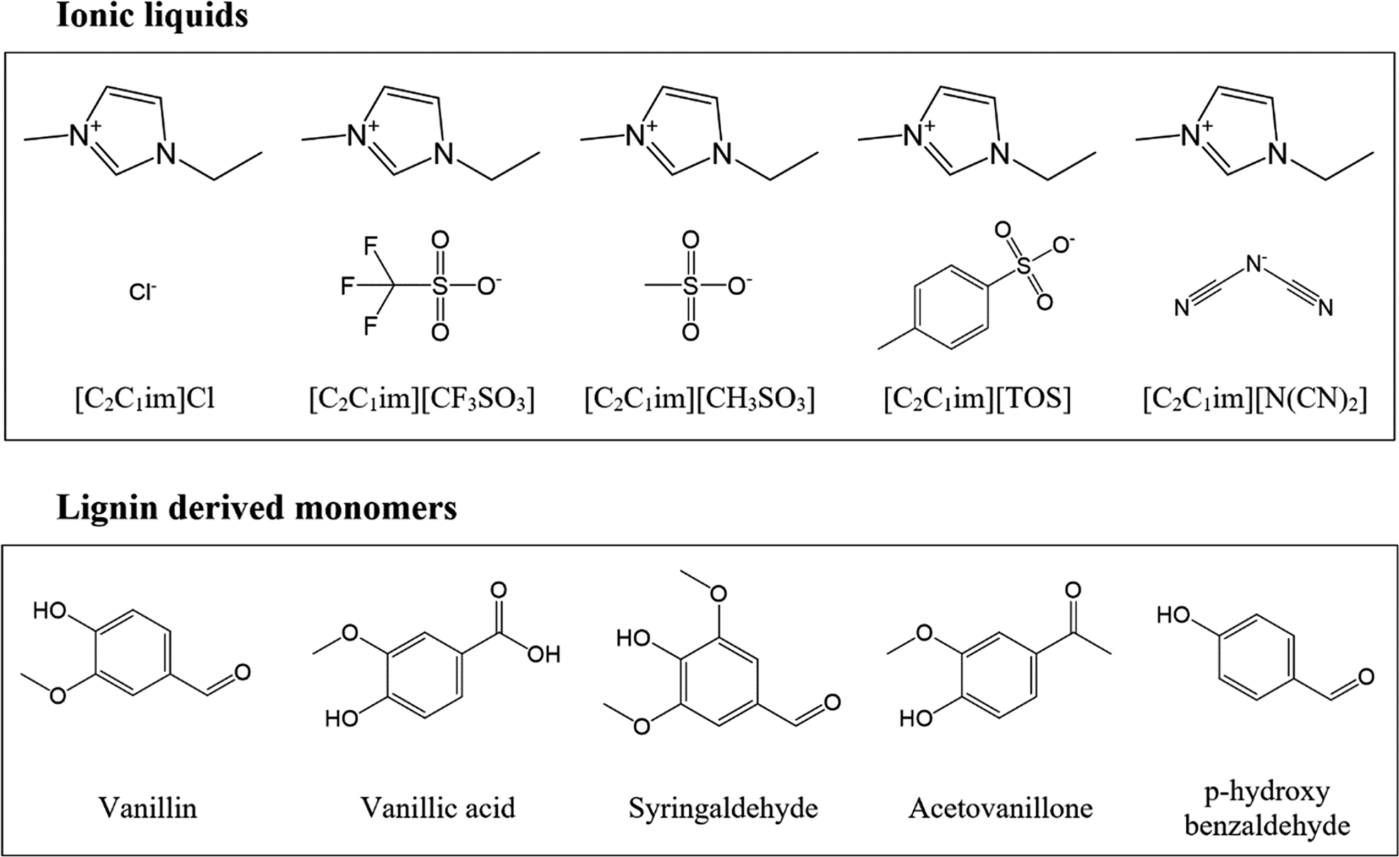
Chemical structures of ionic liquids and lignin-derived monomers used in this work.

The studied polymer–polymer-based ABS is constituted by two polymers, namely, polyethylene glycol (PEG 8000 g·mol^–1^; purum) and sodium polyacrylate (NaPA 8000 g·mol^–1^; 45% in water), both from Sigma-Aldrich. The inorganic salts used as electrolytes were sodium chloride (NaCl) and sodium sulfate (Na_2_SO_4_), both purchased from Sigma-Aldrich with a purity of ≥99%. The ILs (Figure 1) 1-ethyl-3-methylimidazolium chloride ([C_2_C_1_im]Cl), 1-ethyl-3-methylimidazolium triflate ([C_2_C_1_im][CF_3_SO_3_]), 1-ethyl-3-methylimidazolium methane sulfonate ([C_2_C_1_im][CH_3_SO_3_]), 1-ethyl-3-methylimidazolium tosylate ([C_2_C_1_im][TOS]), and 1-ethyl-3-methylimidazolium dicyanamide ([C_2_C_1_im][N(CN)_2_]), used as electrolytes as well, were purchased from Iolitec with a purity of >97%. Among the representative lignin-derived monomers (Figure 1), vanillin (V), acetovanillone (AV), and *p*-hydroxybenzaldehyde (HB) were acquired from Sigma-Aldrich with a purity of >98%, while vanillic acid (VA) and syringaldehyde (SA) were purchased from Acros Organics, both with a purity of >97%.

For HPLC analysis, methanol and formic acid were purchased from Fischer Scientific (HPLC grade) and Carlo Erba (purity ≥ 99%), respectively. Double distilled water was passed through a reverse osmosis system and further treated with a Milli-Q plus 185 apparatus before use. Syringe filters (0.45 μm pore size; Specanalitica, Portugal) and membrane filters (0.22 μm; Sartorius Stedim Biotech, Germany) were applied in filtration steps. Macrosep Advance Centrifugal Devices from PALL were used as ultrafiltration apparatus, while Oasis HLB (200 mg) cartridges from Waters were used in solid-phase extraction experiments.

### Preparation of Polymer–Polymer-Based ABS and Partition of Lignin-Derived Monomers

A polymer–polymer-type ABS composed of PEG 8000 and NaPA 8000 was applied in the partition of lignin-derived monomers. By taking into account the corresponding phase diagrams reported elsewhere,^26^ an extraction point constituted by 15 wt % PEG 8000 + 4.5 wt % NaPA 8000 + 5 wt % electrolyte + 75.5 wt % water (unbuffered) or buffer (at pH 5 or 12) that falls in the biphasic region was chosen for the separation of lignin-derived monomers (0.03 g·L^–1^). For buffered ABS, 0.1 M Na_3_C_6_H_5_O_7_/0.2 M Na_2_HPO_4_ and 0.1 M NaOH/0.2 M Na_2_HPO_4_ were used to adjust the pH to 5 and 12, respectively. The biphasic systems used for lignin monomer partition studies were gravimetrically prepared (with an uncertainty of ±10^–4^ g), stirred, and centrifuged (3500 rpm for 30 min at 298 ± 1 K) to expedite and ensure complete phase separation. The partition experiments were performed in triplicate, and the final concentration of lignin-derived monomers was reported as the average value accompanied by the respective standard deviation. Possible background interferences of the phase-forming electrolytes were considered using blank controls in the absence of lignin-derived monomers, but no discernable interferences were observed. The partition coefficients (*K*) of lignin monomers were calculated as indicated by eq 1:
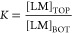
1where [LM]_TOP_ and [LM]_BOT_ represent the lignin monomer concentration in the top and bottom phases, respectively.

### Detection and Quantification of Lignin-Derived Monomers

The lignin monomers were quantified in both top and bottom phases by high-performance liquid chromatography (HPLC). The HPLC apparatus was equipped with an analytical C18 reversed-phase column (250 × 4.6 mm), RP18 CoreShell 5 μm, from SunShell (ChromaNik Technologies, Inc.), and a diode-array detection system (VWR Hitachi Chromaster). The HPLC method for the quantification of these compounds was previously reported^34^ but herein optimized to obtain the best resolution while minimizing the analysis time. The operating conditions included an oven temperature of 40 °C, a flow rate of 1.0 mL·min^–1^, an injection volume of 20 μL, and a binary gradient consisting of solvent A, water–formic acid (98:2 v/v), and solvent B, methanol–water–formic acid (70:28:2 v/v), as follows: 90% isocratic A for 5 min, linear gradient from 90 to 60% A in 10 min, 60% isocratic A for 5 min, linear gradient from 60 to 90% A in 5 min, and 90% isocratic A for 5 min.

The monomers were identified according to their retention time and were quantified on their maximum absorbance wavelengths, ensuring maximum analyte absorbance while simultaneously avoiding solvent interference.^35^ In this context, vanillic acid and *p*-hydroxybenzaldehyde were detected at 290 nm, while vanillin, syringaldehyde, and acetovanillone were detected at 300 nm. Calibration curves for each monomer were determined in the range of 2–20 μg·mL^–1^.

### Separation of Lignin-Derived Monomers by Centrifugal Partition Chromatography

#### Equipment

The separation of lignin monomers was further studied using a CPC system, model CPC-C, from Kromaton Rousselet-Robatel (Annonay, France). The equipment design comprises a pattern of cells interconnected by ducts that are dug into a stainless-steel disk. The rotor consists of 13 associated disks, each containing 64 twin-cells, constituting a total of 832 twin-cells. This so-called twin-cell design contains a restriction in the middle ducts of the canal, creating two superimposed chambers. Therefore, the maximum theoretical liquid stationary phase retention factor is 80% as 20% of the connecting duct volume can only contain the mobile phase. The maximum rotor rotation is 3000 rpm, generating a maximum centrifugal field of ∼1500*g*. Two rotating seals are displayed at the rotor entrance and exit (alternatively designated “head” and “tail”, respectively), which allow a maximum pressure of 7 MPa. The CPC system is connected to a gradient unit with a degasser, an analytical HPLC pump, and a DAD detector (four wavelengths were set for analysis, wherein 300 nm was chosen as a wavelength with reasonable analyte absorbance with less solvent background interference).

#### Conditioning of Equipment

The CPC separations were performed for a system constituted by PEG 8000 + NaPA 8000 + best-performing electrolyte + water (unbuffered)/buffer (at pH 5 or 12). Following their gravimetric preparation and phase separation, both eluents were degasified prior to their introduction to the system to minimize cavitation phenomena. This system was operated in ascending mode. Initially, the rotor was entirely filled with the stationary NaPA 8000-rich bottom phase at a flow rate of 3 mL·min^–1^ at 20 °C to achieve the complete permeation and re-equilibration of the rotor. At least two column volumes of the stationary phase (approximately 80 mL) were pumped in total through the column in this step. Subsequently, the rotational speed was increased to approximately 2000 rpm and the PEG 8000-rich top phase was pumped through the stationary phase until an equilibrium was established within the rotor cells, i.e., when only the mobile phase leaves the column and the signal baseline remains stable. The mobile phase flow rate (1 mL·min^–1^) was initially chosen according to data previously reported of these systems,^33^ but it was later optimized targeting separation. An appropriate flow rate is essential to ensure a steady, continuous flow of the mobile phase, preventing the formation of air pockets in the lines and cells, which significantly affect the absorbance measurements at the detector. The volume of the stationary phase inside the column at an examined flow rate (*V*_S_) was defined as the difference between the total column volume (*V*_C_) and the volume of the stationary phase displaced up to such a flow rate (*V*_SP_), considering the dead volume of the system. Lastly, the maximum theoretical liquid stationary phase retention factor (*S*_f_) was defined as the ratio of the stationary phase volume inside the column at the examined flow rate (*V*_S_) and the column volume (*V*_C_), as represented in eq 2
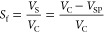
2

In each run, *S*_f_ values of approximately 50% were consistently achieved.

#### Pulse Injection of Samples for Separation of Lignin-Derived Monomers

In the separation trials of the lignin monomeric mixture, the sample loop was initially filled with 10 mL of ABS composed of 15 wt % PEG 8000 + 4.5 wt % NaPA 8000 + 5 wt % best-performing electrolyte + 75.5 wt % water (unbuffered)/buffer (at pH 5 or 12), containing the representative lignin-derived monomers at higher concentrations (0.4 g·L^–1^) than those used in lab-scale experiments (0.03 g·L^–1^) to ensure a strong absorbance signal. Fractions of 1 mL volume were collected during the run and were later analyzed by HPLC as described above. A schematic representation of CPC operation can be found in Figure S1 of the Supporting Information.

### Phase-Forming Constituent Recycling and Isolation of Vanillic Acid

**Figure 2:**
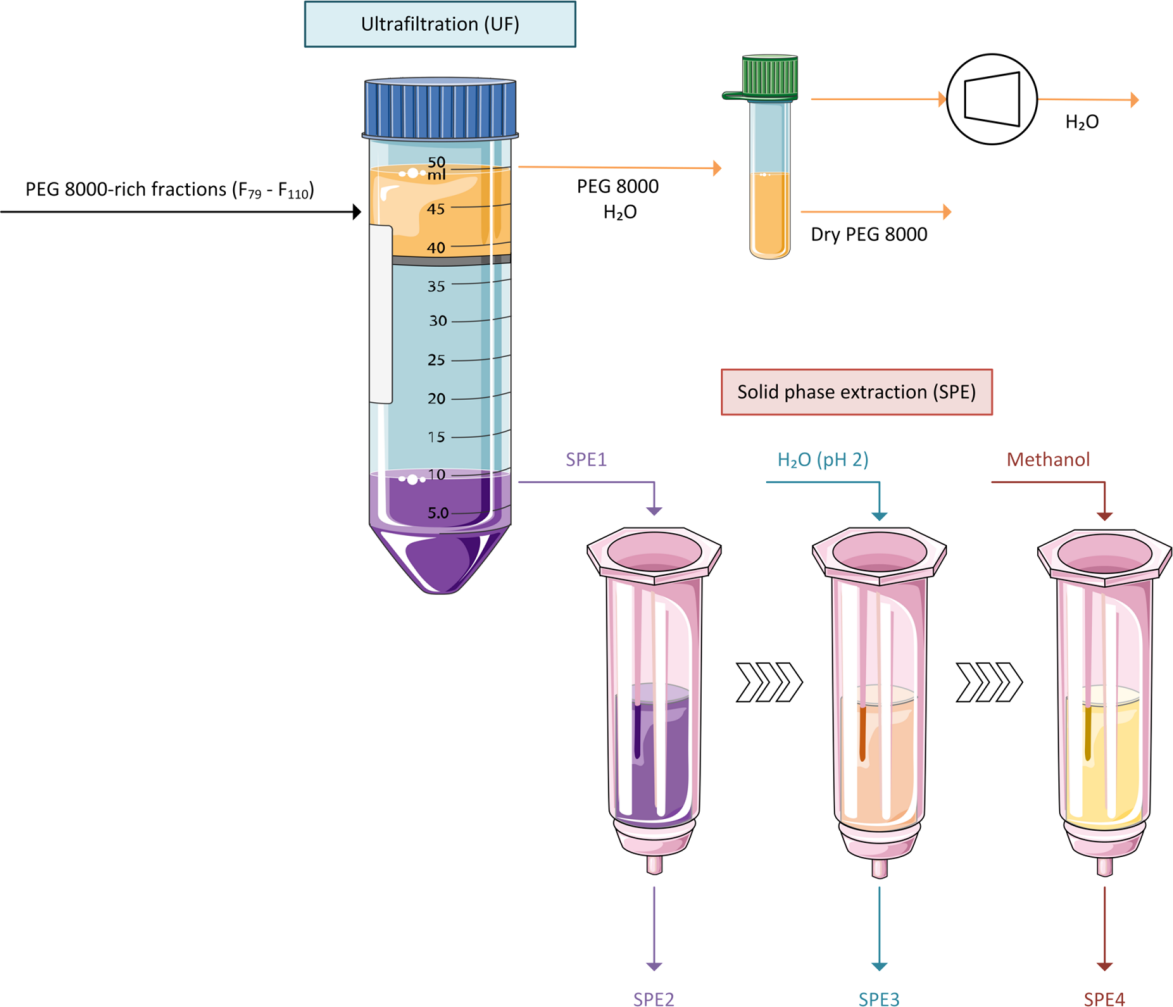
Schematic representation of the selective separation of the phase-forming components and vanillic acid developed in this study.

Collected CPC fractions containing separated vanillic acid and mobile phase (PEG 8000 as a major component and IL electrolyte as a minor component) were further applied in downstream processing by the combination of ultrafiltration and solid-phase extraction steps as represented in Figure 2. The composition of the mobile phase was determined through the liquid–liquid equilibrium data and corresponding binodal curve as described in the Supporting Information.

#### UF and ATR-FTIR Analysis of the Retentate

The combined CPC-recovered fractions were introduced into a Macrosep Advance Centrifugal Device from PALL (MW cutoff: 1 kDa; diameter: 50 mm) and centrifuged at 1496*g*, 25 °C, for 21 h. The retentate enriched in PEG 8000 was subsequently dried, and its purity was ascertained by attenuated total reflectance Fourier-transform infrared (ATR-FTIR) spectroscopy (Tensor 27 FTIR spectrometer, Bruker Co., USA). The FTIR spectra were acquired within a wavenumber ranging from 700 to 4000 cm^–1^, with a resolution of 4 cm^–1^ and 32 scans, and referenced against the neat PEG 8000 polymer. The permeate enriched in vanillic acid and [C_2_C_1_im][N(CN)_2_] was further processed through solid-phase extraction.

#### SPE of the IL-Rich Permeate

The isolation of vanillic acid from the [C_2_C_1_im][N(CN)_2_]-rich permeate was performed by SPE using Oasis HLB cartridges previously washed with methanol. The permeate (SPE1) was initially passed through the packed column, ensuring the adsorption of vanillic acid (and residual amount of other co-eluted lignin monomers) and [C_2_C_1_im][N(CN)_2_] onto the solid phase and releasing SPE2 stream. Acidic water (pH ca. 2) was then used to promote the selective desorption of vanillic acid (and residual amount of other co-eluted lignin monomers) (SPE3), and finally, methanol was eluted to desorb [C_2_C_1_im][N(CN)_2_] (SPE4). All these fractions were collected, and the target compounds were quantified by the HPLC method as previously described.

## Results and Discussion

### Effect of the Electrolyte and pH on the Partition of Lignin-Derived Monomers in PEG-NaPA-Based ABS

The partition of lignin-derived monomers, namely, vanillin, vanillic acid, syringaldehyde, acetovanillone, and *p*-hydroxybenzaldehyde, in PEG-NaPA-based ABS was first investigated. These monomeric compounds are abundant in lignin depolymerization streams,^36−38^ constituting a representative mixture to be examined in this study. PEG-NaPA-based ABS have been previously characterized, and their phase diagram and preferential partition of several electrolytes (inorganic salts and ILs) were determined.^26^ The selection of this specific polymer–polymer-type ABS relies on their capacity for the separation of different biomolecules, particularly phenolic compounds as reported elsewhere.^30,33^ Herein, two inorganic salts (NaCl and Na_2_SO_4_) and five ILs ([C_2_C_1_im]Cl, [C_2_C_1_im][CF_3_SO_3_], [C_2_C_1_im][CH_3_SO_3_], [C_2_C_1_im][TOS], and [C_2_C_1_im][N(CN)_2_]) were applied as electrolytes in PEG-NaPA-based ABS to study the partition of those lignin-derived monomers. The impact of ABS pH on their partition was also studied by screening unbuffered (pH ≈ 7–8) and buffered ABS (pH = 5 and pH = 12). The pH variation is expected to affect the speciation of each lignin monomer (Figure S2 in the SI), which might influence their partition between top and bottom phases of examined PEG-NaPA-based ABS.

**Figure 3:**
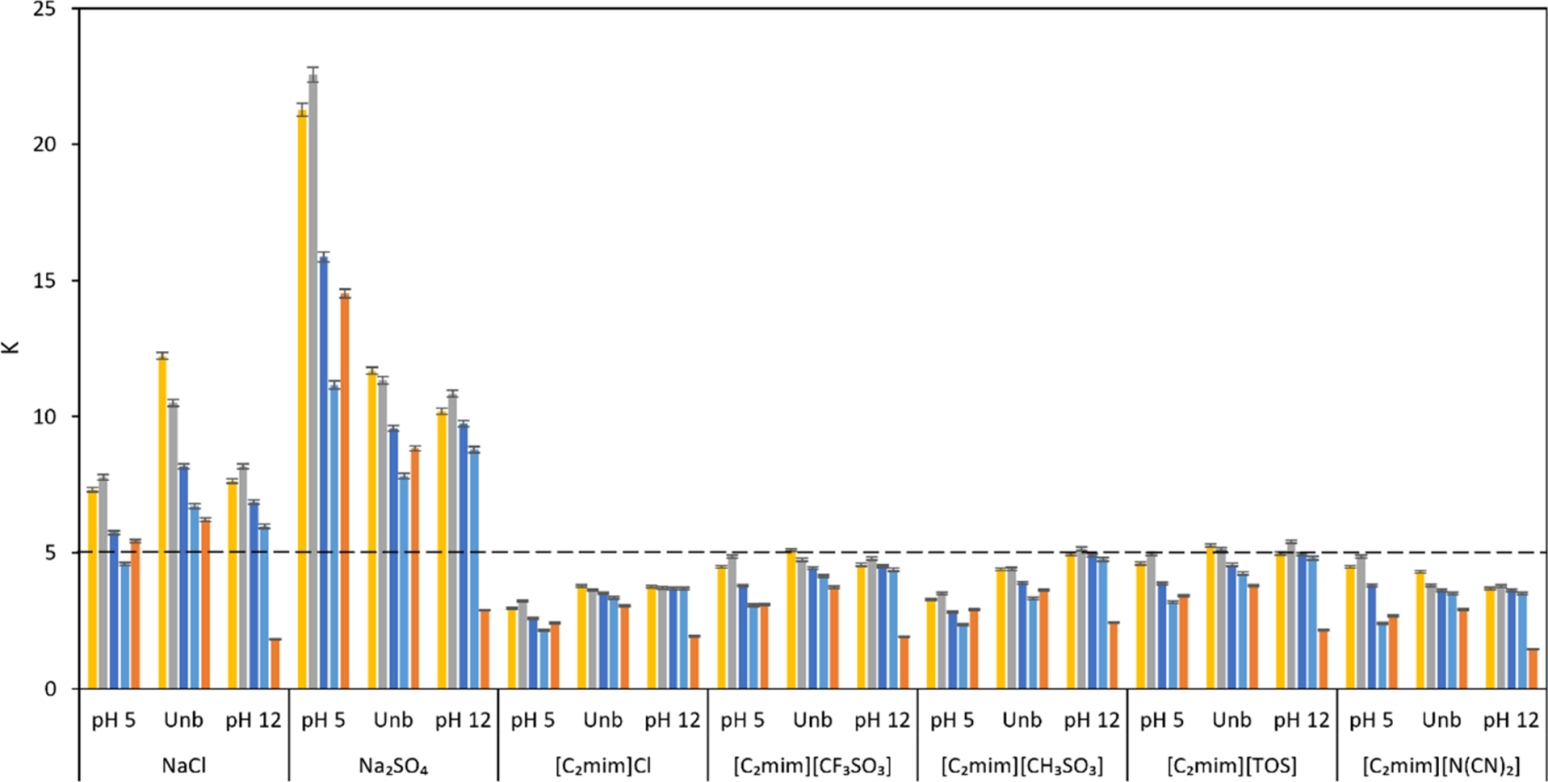
Partition coefficients of acetovanillone (gold bars), syringaldehyde (silver bars), vanillin (dark blue bars), *p*-hydroxybenzaldehyde (light blue bars), and vanillic acid (orange bars) in unbuffered (Unb) and buffered (pH = 5 and pH = 12) PEG 8000 + NaPA 8000 + electrolyte systems. A *K* value of 5 (dashed line) was chosen as the *K* limit to allow CPC separation based on previous knowledge.

The partition coefficients of the different monomers were determined for all systems investigated, being represented in Figure 3.

The analysis of the partition coefficients depicted in Figure 3 allows the identification of some trends. In all examined systems, a partition toward the top PEG-rich phase was observed for all monomers (*K* > 1). The higher hydrophilicity of the bottom NaPA-rich phase enables a higher salt concentration, leading to the salting-out effect of lignin monomers toward the top phase. This effect was more pronounced when using NaCl and Na_2_SO_4_ salts as electrolytes (*K* > 5) than when using ILs (mostly 5 > *K* > 1) either for unbuffered or buffered systems. In the case of Na_2_SO_4_, the slight acidification of the medium (pH 5) increased the partition of lignin monomers to the top phase, while a high alkalinity (pH 12) enabled a decrease in vanillic acid’s partition coefficient in comparison to other compounds. This behavior might be explained by a different speciation exhibited by vanillic acid in contrast to other lignin monomers. At pH 12, vanillic acid is doubly deprotonated (carboxylic and phenolic sites), while the other studied compounds present single deprotonation (phenolic site). This speciation of vanillic acid affects its partitioning in PEG-NaPA-based ABS, which can be seen as an advantage toward its separation from the remaining lignin monomers. However, despite preferable partitioning toward the bottom phase (Table S4), ILs led to a less intense salting out effect of lignin monomers, while the variation of pH did not show a relevant influence on the partition. A similar behavior of vanillic acid partition in a high alkalinity (pH = 12) was also observed, reinforcing the idea that lignin monomer speciation at certain pH values might play an important role in compound migration, particularly regarding vanillic acid. The distinct behavior of aromatic acids in contrast to aromatic aldehydes and ketones at high pH was also reported to be beneficial for adsorption and separation in chromatographic columns.^10^

Bearing in mind the window of *K* values suitable for the CPC technique (*K* < 5), the separation of lignin monomers with the PEG-NaPA-based system assisted by ILs is expected to produce better chromatographic separation and resolution than with inorganic salts. Yet, the *K* values of all lignin monomers still exceeded the upper limit of the theoretical ideal range (0.4 ≤ *K* ≤ 2.5). Moreover, regardless of pH media, the *K* values of vanillin, syringaldehyde, acetovanillone, and *p*-hydroxybenzaldehyde are generally similar, suggesting that co-elution in CPC may occur. In addition, since vanillic acid exhibited *K* values lower than all other compounds at pH 12, it indicates potential for efficient CPC separation of this particular compound from other lignin monomers. For this reason, vanillic acid separation in CPC was targeted and studied with the PEG-NaPA-based system assisted by [C_2_C_1_im][N(CN)_2_] as the electrolyte. This IL provided the highest difference between vanillic acid and other lignin monomers’ *K* values, an important feature to promote separation in CPC.

### Separation of Vanillic Acid Assisted by CPC

**Figure 4:**
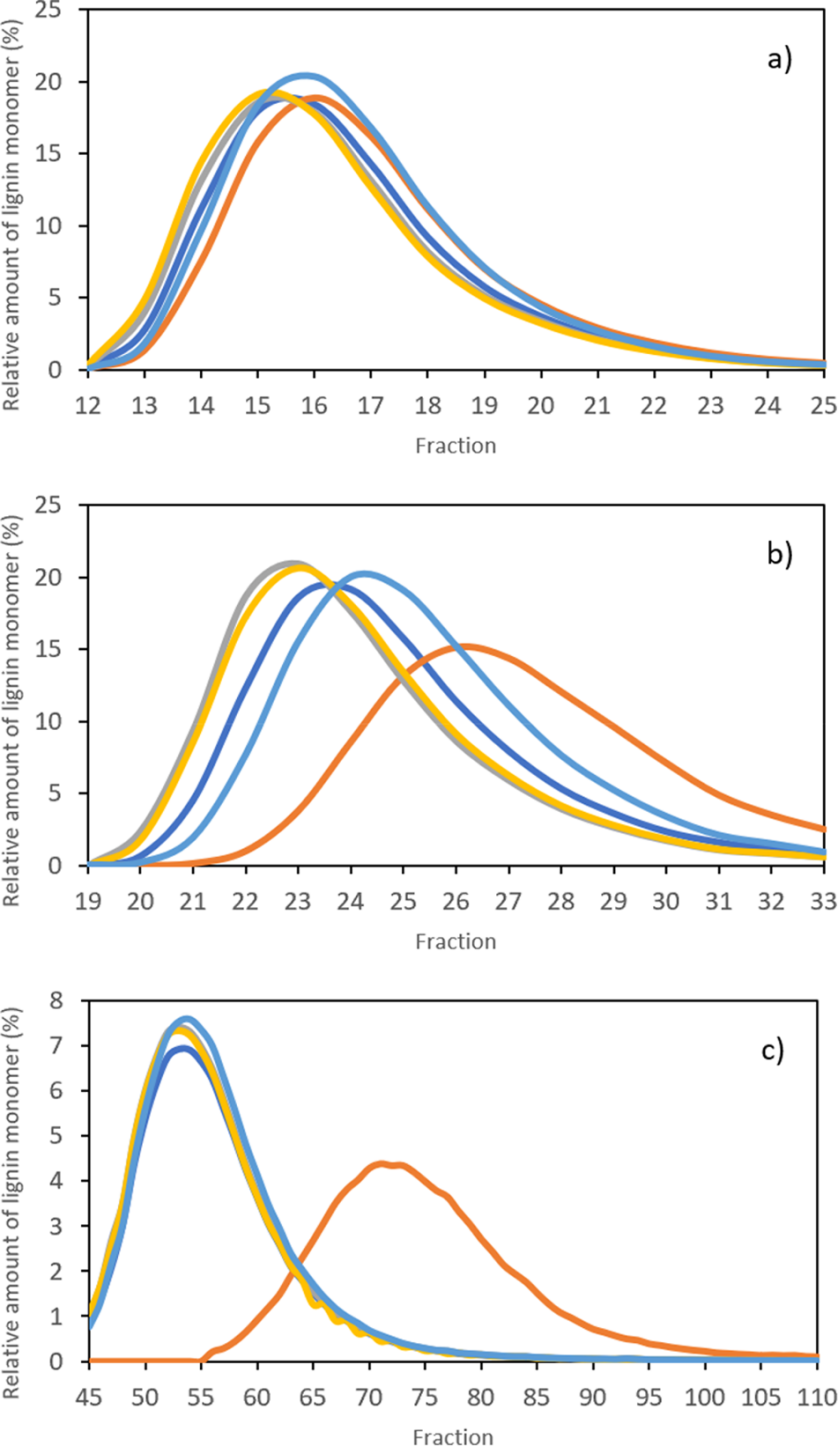
Relative amount of acetovanillone (gold curves), syringaldehyde (silver curves), vanillin (dark blue curves), *p*-hydroxybenzaldehyde (light blue bars), and vanillic acid (orange curves) during CPC separation using the ABS system composed of 15 wt % PEG 8000 + 4.5 wt % NaPA 8000 + 5 wt % [C_2_C_1_im][N(CN)_2_] under different pH conditions: (a) unbuffered (pH ≈ 8), (b) pH 5, and (c) pH 12. Other operating conditions: rotational speed of 2000 rpm; flow rate of 1.5 mL.min^–1^; *S*_f_ ≈ 40%; *P* ≈ 45 MPa; detection wavelength: 300 nm.

A tentative vanillic acid separation through CPC using ABS constituted by PEG 8000 + NaPA 8000 + [C_2_C_1_im][N(CN)_2_] was performed under unbuffered (pH ≈ 8) and buffered conditions (pH 5 and 12). The obtained chromatograms are presented in Figure 4 and present the relative amount of lignin monomer as a function of each collected CPC fraction.

The obtained data show that pH has a significant impact on the separation of the vanillic acid from other lignin monomers. A co-elution of the studied lignin monomers was achieved in the unbuffered system (pH ≈ 8), a consequence of the minor differences between *K* values of lignin monomers under this pH condition in the PEG-NaPA-[C_2_C_1_im][N(CN)_2_] system (Figure 3). Multiple speciation of lignin monomers is induced at pH near 8 (Figure S2 in the SI), leading to inefficient separation. However, as the pH is set to 5, a slight separation of vanillic acid, vanillin, and acetovanillone from *p*-hydroxybenzaldehyde and syringaldehyde was observed. In this case, all lignin monomers are protonated with the exception of vanillic acid, which is in its first deprotonated form. Although *K* values of lignin monomers are distinct at pH 5 (Figure 3), they are not distinct enough to ensure an efficient separation. A high pH (12) in turn enables the complete deprotonation of all lignin monomers, including the formation of a second deprotonation form of vanillic acid. This second deprotonated form increases the polarity of vanillic acid and consequently reduces its partition toward the more hydrophobic PEG-rich phase. The *K* value of vanillic acid decreases sharply in comparison to that of its lignin monomers counterparts (Figure 3), allowing for an improved separation in CPC (Figure 4c). The separation at a flow rate of 1.5 mL·min^–1^ enabled the recovery yield of vanillic acid up to 82.4% with 81.2% of purity (fractions F_64_–F_110_).

**Figure 5:**
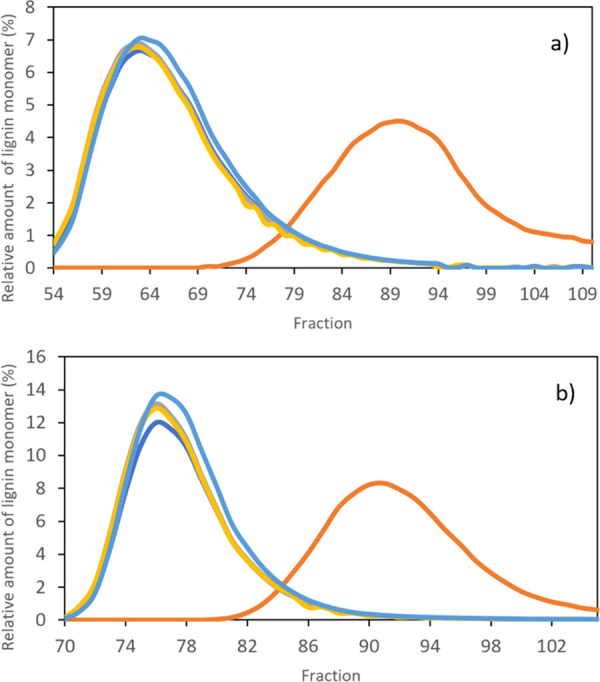
Relative amount of acetovanillone (gold curves), syringaldehyde (silver curves), vanillin (dark blue curves), *p*-hydroxybenzaldehyde (light blue curves), and vanillic acid (orange curves) during CPC separation using the ABS composed of 15 wt % PEG 8000 + 4.5 wt % NaPA 8000 + 5 wt % [C_2_C_1_im][N(CN)_2_] + 75.5 wt % McIlvaine buffer (pH ≈ 12) at different flow rates of the mobile phase: (a) 1.0 and (b) 0.7 mL·min^–1^. Other operating conditions: rotational speed of 2000 rpm; *S*_f_ ≈ 40%; *P* ≈ 45 MPa; detection wavelength: 300 nm.

To maximize the vanillic acid purity, the influence of the CPC flow rate on the resolution of the separation of vanillic acid from the remaining lignin monomeric mixture was subsequently studied. Therefore, at pH 12, two other flow rates (1.0 and 0.7 mL·min^–1^) of the mobile phase were tested besides 1.5 mL·min^–1^ (Figure 4c). The resulting chromatograms are depicted in Figure 5 and, contrasting to Figure 4c, suggest that as the mobile flow rate decreases, the resolution of separation increases. The flow rates of 1.0 and 0.7 mL·min^–1^ enabled the separation and recovery of vanillic acid with purities up to 93.1% (fractions F_79_–F_110_ with 89.4% of recovery yield) and 95.6% (fractions F_85_–F_105_ with 94.3% of recovery yield), respectively.

### Phase-Forming Component Recycling and Isolation of Vanillic Acid

The isolation of vanillic acid from the PEG 8000-rich fractions (F_85_–F_105_) obtained from CPC fractionation at 0.7 mL·min^–1^ flow (optimal conditions) was further addressed by combining two consecutive steps of ultrafiltration and solid-phase extraction. The ultrafiltration envisaged a molecular weight-based separation of vanillic acid (MW: 168 Da) and PEG 8000. While the heavier polymers (MW: ca. 8000 Da) were retained by the ultrafiltration membrane, lower-molecular-weight vanillic acid (MW: 168 Da) and [C_2_C_1_im][N(CN)_2_] (MW: 177 Da) were permeated. This polishing step allowed the successful separation of 92.2% of the vanillic acid present in fractions F_85_–F_105_ resulting from CPC fractionation into the permeate. The obtained retentate was subsequently dried and allowed a successful recovery of 93.6% of PEG 8000 present in F_85_–F_105_ samples. The purity of the recovered PEG 8000 was ascertained by ATR-FTIR spectroscopy, and the obtained FTIR spectra (Figure S4 in the Supporting Information) showed no significant difference between the recovered and neat PEG 8000. Therefore, the recovered polymers can be directly reused in a new purification cycle as previously described for similar systems.^39,40^ Finally, the isolation of vanillic acid from [C_2_C_1_im][N(CN)_2_] (ultrafiltration permeate) was performed by SPE using commercially available column cartridges. This purification step allowed the successful recovery of 94.4% of the vanillic acid present in F_85_–F_105_ samples and the selective recovery of [C_2_C_1_im][N(CN)_2_] for further reuse.

### Overview of the Process

**Figure 6:**
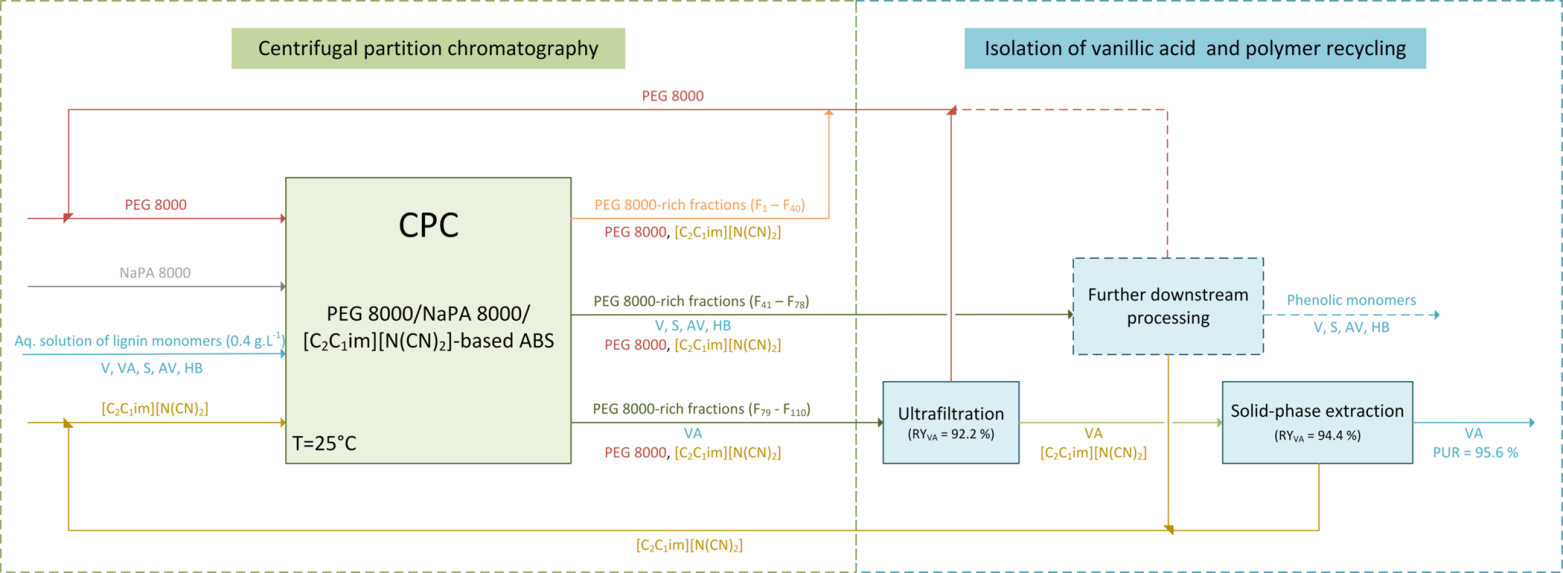
Schematic representation of the envisioned scaled-up process of purification of vanillic acid from other lignin-derived monomers using PEG 8000/NaPA 8000-based ABS with [C_2_C_1_im][N(CN)_2_] as the electrolyte and CPC.

Briefly, an integrated process for the purification of vanillic acid from other lignin-derived monomers was designed in this study, envisaging industrial application. Within a holistic view of the process as depicted in Figure 6, 82.1% recovery yield of initial vanillic acid, with a maximum purity of 95.6%, and simultaneous recycling of applied chemicals (aqueous phase-forming agents) were accomplished. In the literature, one of the best results in vanillic acid separation and purification was achieved with four consecutive steps of ultrafiltration, nanofiltration, and adsorption chromatography using SP700 resin (94.1% of vanillic acid recovery yield, without purity assessment).^41^ The developed separation and downstream process is groundbreaking for vanillic acid purification, offering thus a solid alternative to solvent-consuming adsorption chromatographic runs.

In fact, the developed process based on CPC and ABS is scalable to volumes used in industrial activities, through larger apparatus than the one used in this study, and purchased from equivalent suppliers of CPC equipment. Scaling-up this technology will afford savings in time and materials, reducing costs and reducing the environmental impact (e.g., E-factor) when compared to conventional technologies, such as liquid–liquid and solid–liquid extractions, analytical and preparative column chromatographic methods, and membrane separations, while allowing continuous fractionation.^32,42^ Indeed, the sustainability of the process increases with the application of CPC in continuous mode aligned with the recycling of both mobile and stationary liquid phases. A previous study demonstrated that under two different scenarios, recycling of both liquid phases decreases the carbon footprint of the technology by 36% in comparison to the no-recycling scenario.^33^ The carbon footprint reduction is directly related to the savings of solvent chemicals and water with recirculation of both phases. In another work, the integration of CPC separation at the industrial scale with solvent recycling under continuous mode was already demonstrated by Lorántfy et al. in the separation of active pharmaceutical ingredients, allowing reduction of the total costs of the process.^42^

This study presents fundamental data that will support future application of this technology in real lignin depolymerization streams toward the fractionation of lignin monomers (and oligomers). The simplified operation of CPC is expected to enable easy integration in biorefinery facilities, increasing the efficiency of the downstream processing platform after operations related to biomass fractionation and conversion.

## Conclusions

The present work disclosed a new strategy to recover vanillic acid from a mixture of lignin-derived monomers by coupling ABS with CPC. Specifically, an ABS composed of PEG 8000, NaPA 8000, and [C_2_C_1_im][N(CN)_2_] as the electrolyte, buffered at pH 12, enabled propitious physicochemical properties for the selective separation and isolation of vanillic acid from other lignin-derived monomers mediated by CPC. The phenomenon behind this selectivity toward vanillic acid can be explained by the unique speciation of this compound (double deprotonation) in comparison to other examined lignin-derived monomers (single deprotonation form) under alkaline conditions. The double deprotonation of vanillic acid at pH 12 allowed different interactions with the PEG-rich phase and NaPA-rich phase in contrast to others, leading to selective separation in CPC. This data allows us to conclude that the pH has a substantial effect on the partition and separation of lignin-derived monomers by CPC that must be taken into account in further application of this technology in the separation of compounds from lignin depolymerization streams.

As a proof of concept, the proposed separation and purification process was further upgraded by the selective recovery and high purity of vanillic acid from the PEG 8000-rich fractions through the combination of ultrafiltration and solid-phase extraction steps. In addition, all phase-forming constituents can be further recovered and reused in new separation runs.

## Supporting Information Available

Detailed information regarding the partition coefficient value of the lignin monomers and ILs in PEG-NaPA-based ABS at varying pH values, schematic representation of CPC operation, speciation of lignin-derived monomers, methodology of the determination of phase composition in the selected ABS, and ATR-FTIR data of neat PEG 8000 and recovered PEG 8000 from the ultrafiltration step (PDF)
