# Supplementary material for: Selective Separation of Vanillic Acid from Other Lignin-Derived Monomers Using Centrifugal Partition Chromatography: The Effect of pH
Source: ACS Sustain Chem Eng. 2022 Apr 5;10(15):4913–21. doi: 10.1021/acssuschemeng.1c08082 (PMC9777839; doi:10.1021/acssuschemeng.1c08082)
Supplement: Supplementary file 1 — sc1c08082_si_001.pdf [file sc1c08082_si_001.pdf]

Supporting Information

Selective separation of vanillic acid from other lignin derived monomers using centrifugal partition chromatography: The effect of pH

Inês L. D. Rocha[1], André M. da Costa Lopes[1,2], Sónia P. M. Ventura[1], João A. P. Coutinho[1]*

[1]CICECO – Aveiro Institute of Materials, Department of Chemistry, University of Aveiro, 3810-193 Aveiro, Portugal;

[2]CECOLAB - Collaborative Laboratory Towards Circular Economy, R. Nossa Senhora da Conceição, 3405-155 Oliveira do Hospital, Portugal

*jcoutinho@ua.pt

Number of Pages: 9

Number of Tables: 6

Number of Figures: 4

TABLE OF CONTENTS

PARTITION COEFFICIENTS 3
CENTRIFUGAL PARTITION CHROMATOGRAPHY (CPC) OPERATION 5
SPECIATION OF LIGNIN DERIVED MONOMERS 6
DETERMINATION OF PHASE COMPOSITION 7
ATR-FTIR DATA 9
REFERENCES 9

PARTITION COEFFICIENTS

Table S1. Partition coefficient of lignin monomers (K_{LM}), namely vanillin (V), vanillic acid (VA), syringaldehyde (SA), acetovanillone (AV) and p-hydroxybenzaldehyde (HB), using inorganic salts or ILs as electrolytes in unbuffered PEG-NaPA-based ABS. The pH values of each ABS are also given.

**Electrolyte**	**$K_V \pm \sigma$**	**$K_{VA} \pm \sigma$**	**$K_{SA} \pm \sigma$**	**$K_{AV} \pm \sigma$**	**$K_{HB} \pm \sigma$**	**pH (± 0.01)**	
						Top	Bottom
NaCl	7.5 ± 1.3	11 ± 1.4	5.4 ± 0.90	8.7 ± 0.80	6.0 ± 1.3	6.64	7.23
Na_2SO_4	19 ± 1.5	10 ± 0.8	7.7 ± 1.3	14 ± 1.2	11 ± 1.2	6.91	7.61
[C_2C_1im]Cl	3.5 ± 0.90	24 ± 1.1	8.1 ± 1.3	3.7 ± 1.2	4.4 ± 1.7	7.17	8.09
[C_2C_1im][CF_3SO_3]	4.6 ± 1.2	2.8 ± 1.2	5.3 ± 1.4	5.0 ± 1.4	4.5 ± 1.4	8.00	8.33
[C_2C_1im] [CH_3SO_3]	4.2 ± 1.2	4.5 ± 0.90	4.1 ± 1.1	3.5 ± 0.90	2.7 ± 1.2	7.93	8.34
[C_2C_1im][TOS]	4.0 ± 0.90	3.9 ± 1.1	7.3 ± 1.6	5.4 ± 1.1	5.7 ± 1.5	8.02	8.27
[C_2C_1im][$N(CN)_2$]	4.5 ± 1.1	4.0 ± 0.90	7.0 ± 1.1	4.3 ± 1.1	4.5 ± 1.2	7.90	8.39

Table S2. Partition coefficient of lignin monomers (K_{LM}), namely vanillin (V), vanillic acid (VA), syringaldehyde (SA), acetovanillone (AV) and p-hydroxybenzaldehyde (HB), using inorganic salts or ILs as electrolytes in buffered PEG-NaPA-based ABS at pH 5. The pH values of each ABS are also given.

**Electrolyte**	**$K_V \pm \sigma$**	**$K_{VA} \pm \sigma$**	**$K_{SA} \pm \sigma$**	**$K_{AV} \pm \sigma$**	**$K_{HB} \pm \sigma$**	**pH (± 0.01)**	
						Top	Bottom
NaCl	5.7 ± 1.3	5.4 ± 1.2	7.8 ± 0.80	7.3 ± 1.2	4.6 ± 1.5	4.87	5.01
Na_2SO_4	16 ± 1.2	15 ± 0.90	23 ± 1.2	21 ± 1.2	11 ± 1.6	5.50	5.44
[C_2C_1im]Cl	2.6 ± 1.2	2.4 ± 1.1	3.2 ± 1.2	3.0 ± 1.0	2.2 ± 1.3	5.50	5.67
[C_2C_1im][CF_3SO_3]	3.8 ± 1.0	3.1 ± 1.1	4.9 ± 1.4	4.5 ± 0.80	3.1 ± 1.2	5.78	5.76
[C_2C_1im] [CH_3SO_3]	2.8 ± 1.3	2.9 ± 1.4	3.5 ± 1.3	3.3 ± 0.80	2.4 ± 1.4	5.73	5.82
[C_2C_1im][TOS]	3.9 ± 0.90	3.4 ± 1.2	5.0 ± 1.1	4.6 ± 1.1	3.2 ± 1.2	5.79	5.73
[C_2C_1im][$N(CN)_2$]	3.8 ± 1.1	2.7 ± 1.0	4.9 ± 1.0	4.5 ± 1.3	3.1 ± 1.1	5.74	5.83

Table S3. Partition coefficient of lignin monomers (K_{LM}), namely vanillin (V), vanillic acid (VA), syringaldehyde (SA), acetovanillone (AV) and p-hydroxybenzaldehyde (HB), using inorganic salts or ILs as electrolytes in buffered PEG-NaPA-based ABS at pH 12. The pH values of each ABS are also given.

Electrolyte	$K_V \pm \sigma$	$K_{VA} \pm \sigma$	$K_{SA} \pm \sigma$	$K_{AV} \pm \sigma$	$K_{HB} \pm \sigma$	pH (± 0.01)	
						Top	Bottom
NaCl	6.9 ± 1.0	1.8 ± 1.2	8.2 ± 0.90	7.6 ± 1.5	6.0 ± 1.3	11.85	11.94
Na_2SO_4	9.7 ± 1.1	2.9 ± 1.1	11 ± 1.0	10 ± 1.2	8.8 ± 1.3	12.00	12.02
$[C_2C_1im]Cl$	3.7 ± 0.90	1.9 ± 1.2	3.7 ± 1.2	3.7 ± 1.1	3.7 ± 1.1	12.07	11.85
$[C_2C_1im][CF_3SO_3]$	4.5 ± 1.4	1.9 ± 0.90	4.8 ± 1.2	4.6 ± 1.2	4.4 ± 1.0	12.02	11.92
$[C_2C_1im]$ $[CH_3SO_3]$	4.9 ± 1.1	2.4 ± 1.3	5.1 ± 1.1	5.0 ± 1.3	4.8 ± 0.90	11.51	11.60
$[C_2C_1im][TOS]$	5.0 ± 1.2	22 ± 1.1	5.4 ± 0.90	5.0 ± 0.80	4.8 ± 1.2	12.17	12.15
$[C_2C_1im][N(CN)_2]$	3.6 ± 0.90	1.5 ± 1.4	3.8 ± 1.3	3.7 ± 1.2	3.5 ± 1.1	12.13	12.29

Table S4. Partition coefficient of the ILs (K_{IL}) as electrolytes in PEG-NaPA-based ABS.[1]

Electrolyte	$K_{IL} \pm \sigma$ (%)
$[C_2C_1im]Cl$	0.77 ± 0.01
$[C_2C_1im][CF_3SO_3]$	0.44 ± 0.01
$[C_2C_1im][CH_3SO_3]$	0.34 ± 0.02
$[C_2C_1im][TOS]$	0.69 ± 0.04
$[C_2C_1im][N(CN)_2]$	0.98 ± 0.03

CENTRIFUGAL PARTITION CHROMATOGRAPHY (CPC) OPERATION

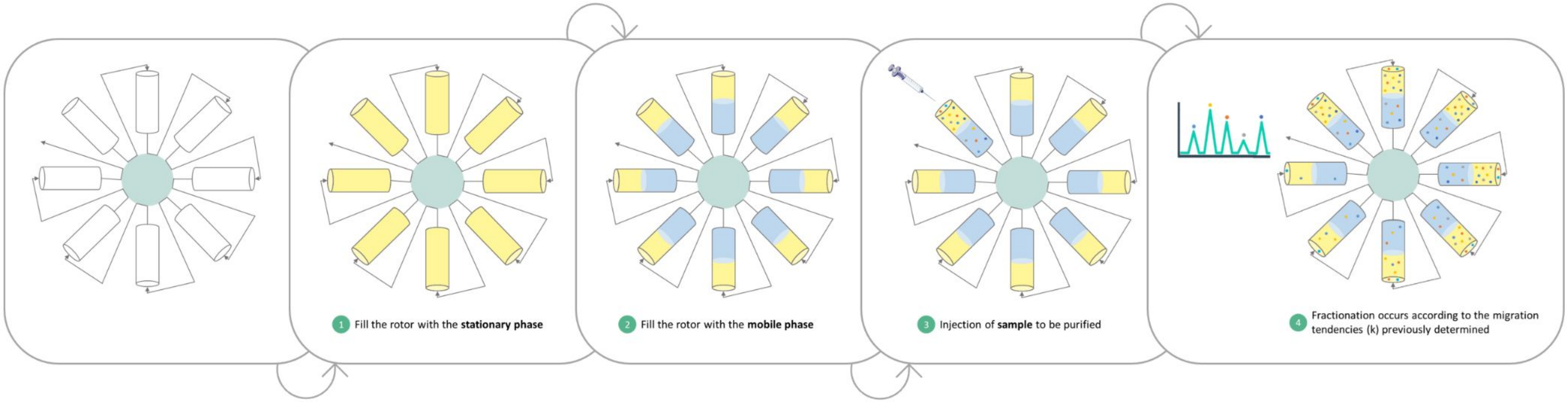

Figure S1. Schematic representation of centrifugal partition chromatography (CPC) operation

SPECIATION OF LIGNIN DERIVED MONOMERS

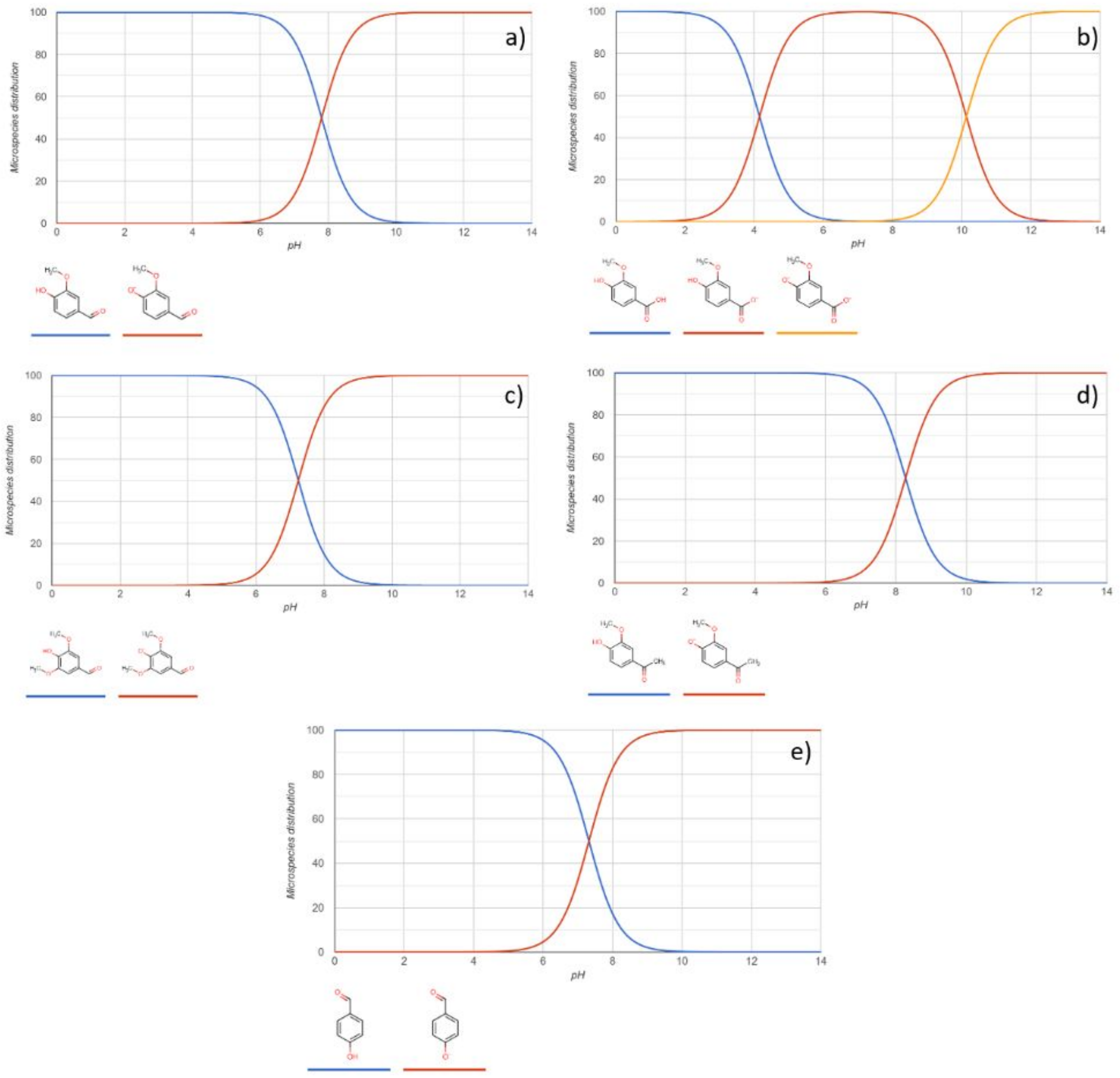

Figure S2. Speciation of a) vanillin, b) vanillic acid, c) syringaldehyde, d) acetovanillone and d) p-hydroxybenzaldehyde as a function of pH (ChemSpider database accessed at 17/09/2021).

DETERMINATION OF PHASE COMPOSITION

The composition of the mobile phase, corresponding to the top phase of the binary mixture at selected extraction point (15 wt% PEG 8000 + 4.5 wt% NaPA 8000 + 5 wt% IL + 75.5 wt% H_2O), was calculated from the equilibrium data determined by cloud titration (Table S5 in supporting information) to which was fitted the binodal curve, as calculated by the Merchuk equation (eq. (3)).

$$[\text{PEG } 8000] = \text{A} \cdot \exp\left[(\text{B} \cdot [\text{NaPA } 8000]^{0.5}) - (\text{C} \cdot [\text{NaPA } 8000]^{3})\right] \quad (3)$$

where [PEG 8000] and [NaPA 8000] represent the weight percentages of PEG 8000 and NaPA 8000, respectively. A, B and C are constants obtained by the regression of the experimental data (Table S5). A representation of the tie-line corresponding to the selected ABS can be found in Figure S3 of the supporting information. The composition of its respective top and bottom phases may be found in Table S6.

Table S5. Parameters obtained through the Merchuk equation with the respective standard deviations (σ) and correlation factors (R^2) along with the weight fraction data (w) for the quaternary systems composed of NaPA 8000 (1) + PEG 8000 (2) + H_2O + $[C_2C_1im][N(CN)_2]^2$.

A ± σ= 38.22 ± 0.99
B ± σ= -9.11 ± 0.02
C ± σ= $2.8 \cdot 10^{-4} \pm 6.0 \cdot 10^{-5}$

R^2=0.9949

**100 w_1**	**100 w_2**
0.50	20.80
0.69	18.15
0.81	16.73
0.89	15.61
1.05	14.53
1.24	13.38
1.43	12.66
1.54	11.93
1.75	11.44
1.95	10.80
2.09	10.36
2.33	9.93
2.42	9.42
2.52	9.07
2.68	8.81

2.75	8.64
2.88	8.34
3.00	8.10
3.04	7,96
3.06	7.76
3.07	7.59
3.33	7.35
3.37	7.12
3.47	6.98
3.42	6.94
3.51	6.52
3.62	6.50
3.71	6.45
3.80	6.11
3.93	6.10
4.01	6.00
4.15	5.73

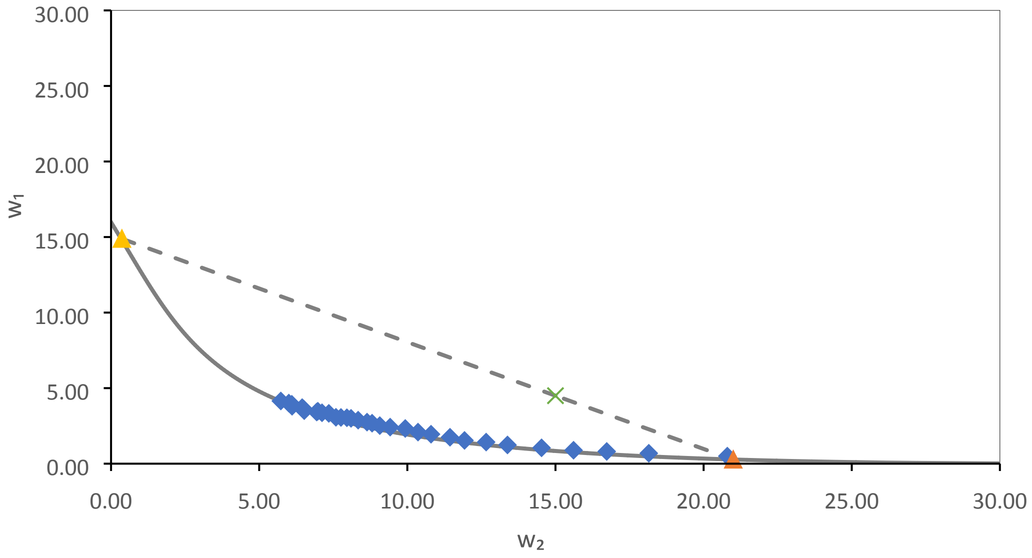

Figure S3. Phase diagrams of the polymeric ABS containing 5.0 wt% of [C_2C_1im][N(CN)$_2$]. Binodal curves fitted to experimental data determined by cloud point titration (◆), using Eq. (3), are represented in full line. The chosen extraction point, constituted by 15 wt% of PEG 8000 + 4.5 wt% of NaPA 8000 + 5.0 wt% of [C_2C_1im][N(CN)$_2$] + 75.5 wt% of H_2O (■), in addition to the composition of the respective top (▶) and bottom (▶) phases, are represented in this figure as well. The corresponding tie line is represented by the dashed line.

Table S6. Top and bottom phase compositions corresponding to the selected extraction point, constituted by 15 wt% of PEG 8000 + 4.5 wt% of NaPA 8000 + 5 wt% of $[C_2C_1im][N(CN)_2]$ + 75.5 wt% of H_2O.

Phase forming compound	Top phase wt%	Bottom phase wt%
PEG 8000	21.00	0.37
NaPA 8000	0.27	14.89
$[C_2C_1im][N(CN)_2]$	5.00	5.00
H_2O	73.73	79.75

ATR-FTIR DATA

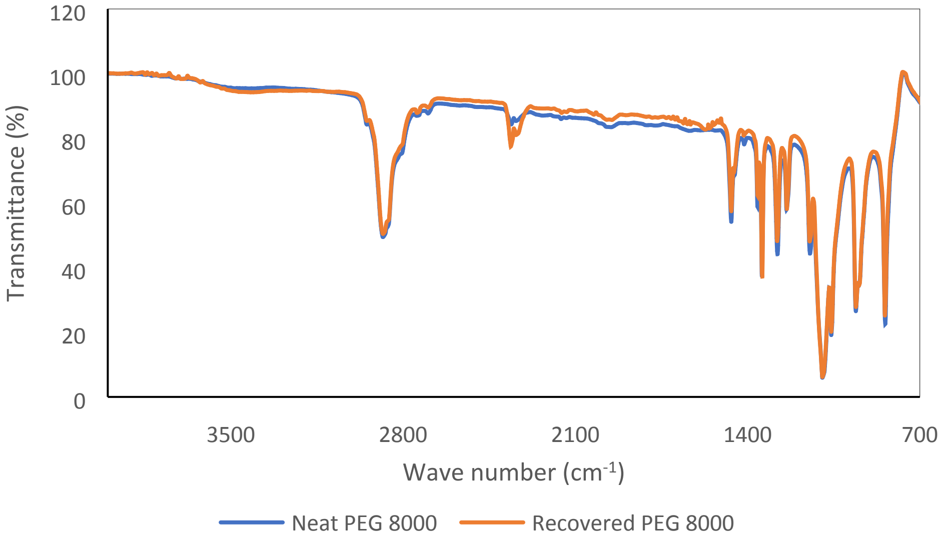

Figure S4. ATR-FTIR spectra of neat PEG 8000 and recovered PEG 8000 from the ultrafiltration step.

REFERENCES

(1) Santos, J. H. P. M.; Almeida, M. R.; Martins, C. I. R.; Dias, A. C. R. V.; Freire, M. G.; Coutinho, J. A. P.; Ventura, S. P. M. Separation of Phenolic Compounds by Centrifugal Partition Chromatography. *Green Chem.* **2018**, *20* (8), 1906–1916. https://doi.org/10.1039/c8gc00179k.

(2) Santos, J. H. P. M.; E Silva, F. A.; Coutinho, J. A. P.; Ventura, S. P. M.; Pessoa, A. Ionic Liquids as a Novel Class of Electrolytes in Polymeric Aqueous Biphasic Systems. *Process Biochem.* **2015**, *50* (4), 661–668. https://doi.org/10.1016/j.procbio.2015.02.001.
